# Functional consequences of somatic mutations in cancer using protein pocket-based prioritization approach

**DOI:** 10.1186/s13073-014-0081-7

**Published:** 2014-10-14

**Authors:** Huy Vuong, Feixiong Cheng, Chen-Ching Lin, Zhongming Zhao

**Affiliations:** Department of Biomedical Informatics, Vanderbilt University School of Medicine, 2525 West End Avenue, Suite 600, Nashville, TN 37203 USA; Department of Cancer Biology, Vanderbilt University School of Medicine, Nashville, TN 37232 USA; Vanderbilt-Ingram Cancer Center, Vanderbilt University Medical Center, Nashville, TN 37232 USA; Center for Quantitative Sciences, Vanderbilt University Medical Center, Nashville, TN 37232 USA

## Abstract

**Background:**

Recently, a number of large-scale cancer genome sequencing projects have generated a large volume of somatic mutations; however, identifying the functional consequences and roles of somatic mutations in tumorigenesis remains a major challenge. Researchers have identified that protein pocket regions play critical roles in the interaction of proteins with small molecules, enzymes, and nucleic acid. As such, investigating the features of somatic mutations in protein pocket regions provides a promising approach to identifying new genotype-phenotype relationships in cancer.

**Methods:**

In this study, we developed a protein pocket-based computational approach to uncover the functional consequences of somatic mutations in cancer. We mapped 1.2 million somatic mutations across 36 cancer types from the COSMIC database and The Cancer Genome Atlas (TCGA) onto the protein pocket regions of over 5,000 protein three-dimensional structures. We further integrated cancer cell line mutation profiles and drug pharmacological data from the Cancer Cell Line Encyclopedia (CCLE) onto protein pocket regions in order to identify putative biomarkers for anticancer drug responses.

**Results:**

We found that genes harboring protein pocket somatic mutations were significantly enriched in cancer driver genes. Furthermore, genes harboring pocket somatic mutations tended to be highly co-expressed in a co-expressed protein interaction network. Using a statistical framework, we identified four putative cancer genes (*RWDD1*, *NCF1*, *PLEK*, and *VAV3*), whose expression profiles were associated with overall poor survival rates in melanoma, lung, or colorectal cancer patients. Finally, genes harboring protein pocket mutations were more likely to be drug-sensitive or drug-resistant. In a case study, we illustrated that the *BAX* gene was associated with the sensitivity of three anticancer drugs (midostaurin, vinorelbine, and tipifarnib).

**Conclusions:**

This study provides novel insights into the functional consequences of somatic mutations during tumorigenesis and for anticancer drug responses. The computational approach used might be beneficial to the study of somatic mutations in the era of cancer precision medicine.

**Electronic supplementary material:**

The online version of this article (doi:10.1186/s13073-014-0081-7) contains supplementary material, which is available to authorized users.

## Background

A major goal in cancer genomics is to understand the genotype-phenotype relationship among genetic alterations, tumorigenesis, tumor progression, and anticancer drug responses. Several large-scale cancer genomic projects, such as The Cancer Genome Atlas (TCGA) and the International Cancer Genome Consortium (ICGC), have generated massive amounts of cancer genomic data, providing us with unprecedented opportunities to study the relationship between genetic alterations and specific cancer phenotypes [1,2]. However, the majority of somatic mutations detected in cancer are ‘passenger’ rather than ‘driver’ mutations [3]. Identifying the functional consequences of somatic mutations during tumorigenesis and tumor progression remains a monumental challenge to cancer genomic studies.

As of April 2014, approximately 100,000 three-dimensional (3D) structures have been included in the Protein Data Bank (PDB) database [4], including approximately 22,000 human protein and nucleic acid 3D structures [5]. Protein structure and function are closely related, especially in the case of protein pockets, which are local regions that perform a variety of critical functions in cells, including binding with small molecules, enzymes, and nucleic acids [6]. Thus, protein pockets are central, structural units in proteins that provide site-specific information as to how a protein interacts with small molecules [7]. With an increasing amount of both protein structural data in the PDB database and somatic mutation data generated by next-generation sequencing (NGS) experiments, the integration of protein structural information and large-scale somatic mutations offers an alternative, promising approach to uncovering functionally important somatic mutations in cancer. Several recent studies have demonstrated that disease-causing mutations commonly alter protein folding, protein stability, and protein-protein interactions (PPIs), often leading to new disease phenotypes [8-20]. Espinosa *et al.* [21] proposed a predictor, InCa (Index of Carcinogenicity) that integrates somatic mutation profiles from the Catalogue of Somatic Mutations in Cancer (COSMIC) database and the neutral mutations from the 1000 Genomes project into protein structure and interaction interface information. Using these data, they developed the InCa classifier model to predict cancer-related mutations with 83% specificity and 77% sensitivity. Ryslik *et al.* [13] developed an approach, *SpacePAC* (Spatial Protein Amino acid Clustering), to identify mutational clustering by directly considering the protein tertiary structure in 3D space. Utilizing the mutational data from the COSMIC and protein structure information from the PDB, they identified several novel mutation clusters using *SpacePAC*. Ghersi and Singh [22] reported that residues located in nucleic acids, small molecules, ions, and peptide binding sites are more likely to be affected by somatic mutations than other residues. Furthermore, protein pocket regions play an important functional role in drug design and development through the ligand-dependent mechanism that affects small molecule binding [23]. For example, several independent research groups found that the presence of mutations in the *EGFR* gene (point mutations in exon 21 or deletions in exon 19) could activate the gene by altering the ATP binding site, ultimately leading to an enhancement of the gefitinib response [24,25]. However, it has been debated whether mutations in the protein pocket regions alter protein functions through the ligand-independent mechanisms [26].

In this study, we proposed a computational approach to investigate 1.2 million somatic mutations across 36 cancer types from the COSMIC database and TCGA onto the protein pocket regions of over 5,000 3D protein structures. We seek to answer two overarching questions: (1) Do the somatic mutations located in protein pocket regions tend to be actionable mutations? and (2) are those specific mutations more likely to be involved in tumorigenesis and anticancer drug responses? Through our systematic analyses, we showed that genes harboring protein pocket somatic mutations tend to be cancer genes. Furthermore, genes harboring protein pocket somatic mutations tend to be highly co-expressed in the co-expressed protein interaction network (CePIN). We identified four putative cancer genes (*RWDD1*, *NCF1*, *PLEK*, and *VAV3*), whose gene expression profiles were associated with overall poor survival rates in melanoma, lung, or colorectal cancer patients. Moreover, by integrating cancer cell line mutations and drug pharmacological data from the Cancer Cell Line Encyclopedia (CCLE), we showed that those genes harboring protein pocket mutations are enriched in drug sensitivity genes. In a case study, we demonstrated that a *BAX* gene with pocket mutations was significantly associated with the drug responses of three anticancer drugs. Collectively, we unveiled that somatic mutations in protein pocket regions tend to be functionally important during tumorigenesis and sensitive to anticancer drug responses. In summary, the protein pocket-based prioritization of somatic mutations provides a promising approach to uncover the putative cancer drivers and anticancer drug response biomarkers in the post-genomic era for cancer precision medicine.

## Methods

### Protein pocket information

We downloaded a list of 5,371 PDB structures with protein pocket information from the Center for the Study of Systems Biology website at Georgia Institute of Technology [27,28]. This library contained only non-redundant, monomeric, single-domain protein structures, measuring 40 to 250 residues in length and registering less than 35% global pair-wise sequence identity. A pocket detection algorithm called LPC (ligand protein contact) was applied to the PDB dataset to generate a set of 20,414 ligand-binding protein pockets whose coordinates were given in each PDB file under the header ‘PKT’, which is an abbreviation for ‘pocket’ [28]. We first parsed out all 5,371 PDB files to obtain pocket residues and their PDB coordinates under the PKT header. Then, we used information from the Structure Integration with Function, Taxonomy, and Sequence (SIFTS) database [29] to translate the PDB coordinates into UniProt coordinates. As of April 2014, approximately 100,000 3D structures have been added to the PDB database, including approximately 22,000 human protein and nucleic acid structures (22%). Since we only focused on mapping somatic mutations onto human protein structures, we filtered out proteins whose organisms were not human, using human protein information from BioMart [30] to obtain a high-quality list of 606 human proteins. We further removed titin (Uniprot ID: Q8WZ42), which is encoded by the longest human gene, *TTN*, but has not yet been detected as cancer-related [31].

### Collection and preparation of somatic mutations

**Figure 1:**
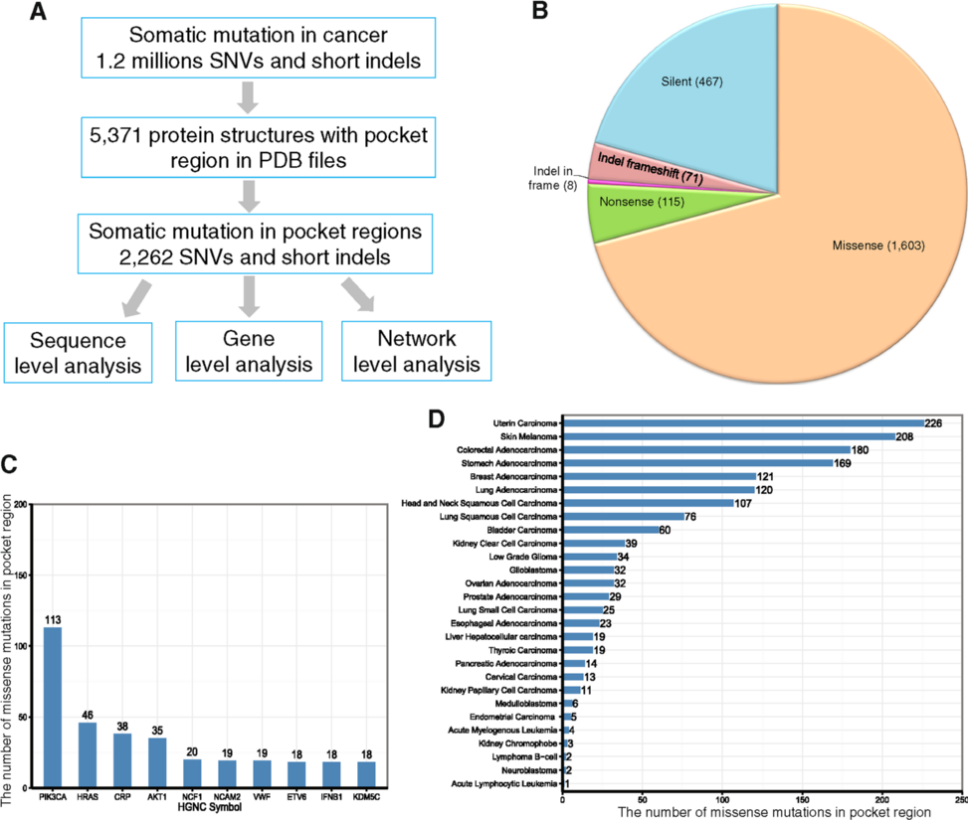
**Computational workflow and general summary. (A)** The protein pocket-based integrative analysis workflow. **(B)** The distribution of protein pocket mutations by mutation types. **(C)** The number of missense mutations in the pocket regions of the top 10 frequently mutated genes. **(D)** Distribution of the number of missense mutations in the pocket regions in 28 cancer types. The detailed data are provided in Additional file 1: Table S1.

The somatic mutation data set was downloaded from Dr. Elledge’s laboratory website at Harvard University [32,33], which contained 1,195,223 somatic mutations from 8,207 tumor samples across 30 tumor types. Somatic mutations with wild-type amino acids in their mutations that were identical to the pocket residues (both residue names and UniProt coordinates) were mapped onto the pocket regions of a total of 606 human proteins. This mapping procedure yielded a total of 3,256 pocket region mutations in 369 unique human proteins. Because identical mutations (defined as having the same wild-type amino acid, alternative amino acid, and UniProt coordinates) could occur in multiple pockets, we removed those duplicated mutations (994 mutations total). The final list of pocket mutations contained 2,262 unique mutations. Among them, there were 1,603 missense mutations, 115 nonsense mutations, 467 silent mutation, 79 short insertions/deletions (indels), and one complex missense (see Additional file 1: Table S1 and Figure 1B). We retained missense mutations in order to predict putative cancer genes in our follow-up statistical analyses.

### Collection of cancer-associated genes

We collected a large number of cancer-associated genes from several publicly available resources. First, a total of 487 genes were downloaded from the Cancer Gene Census [34] (accessed on 10 July 2013, denoted as ‘CGC genes’). CGC genes are well-curated and have been widely used as a reference gene set in many cancer-related projects [35]. Second, we collected 125 cancer driver genes from Vogelstein *et al.* [3]. Finally, we used the 4,050 cancer-associated genes from a previous study [36]. These 4,050 cancer-associated genes were selected based on expert curation or annotation information from the main public databases, experimentally validated cancer genes, and cancer-mutated genes from recent cancer whole exome and whole genome sequencing projects [36]. These genes were used as cancer-associated genes to complement with other carefully curated cancer genes.

### Construction of a high-quality protein interaction network

We downloaded human PPI data from two resources: InnateDB [37] and the Protein Interaction Network Analysis (PINA) platform [38] (accessed on 1 May 2013). Briefly, InnateDB contains more than 196,000 experimentally validated molecular interactions from human, mouse, and bovine models. PINA (v2.0) is a comprehensive PPI database that integrates six large-scale public databases: IntAct, MINT, BioGRID, DIP, HPRD, and MIPS MPact. Similar to our previous work [36,39], in this study, we used only PPI pairs that were experimentally validated through a well-defined experimental protocol. We used two data cleaning steps. First, all protein-coding genes were annotated with Entrez Gene IDs, chromosome location, and the gene symbol from the NCBI database. Second, duplicated or self-loop PPI pairs were removed. After undertaking the data cleaning process, we obtained a total of 113,472 unique PPI binary pairs among 13,579 proteins.

### Construction of a co-expressed protein interaction network

We calculated the gene co-expression correlation for all gene-gene pairs using the microarray gene expression data of 126 normal tissues [40]. The quantile normalization method was used to normalize expression values at the probe level. We then computed the Pearson correlation coefficient (PCC) based on the normalized expression values. Finally, we mapped the PCC value of all protein-protein pairs encoded by genes in the above microarray gene expression data set to the abovementioned PIN to build CePIN based on a previous study [41].

### Somatic mutations of the cancer cell lines

We downloaded the somatic mutations of 1,651 genes across approximately 1,000 cancer cell lines from the CCLE database (accessed on 1 August 2013) at the website [42]. All mutations were determined through targeted, massive parallel sequencing, as described in a previous study [43].

### Drug pharmacological data

We downloaded drug pharmacological data from two previous studies [43,44]. First, Barretina *et al.* [43] tested the pharmacological profiles of 24 anticancer drugs across 504 cell lines. Second, Garnett *et al.* [44] assayed 48,178 drug-cell line combinations with a range of 275 to 507 cell lines per drug and 130 anticancer drugs. The pharmacological data across cell lines, based on the half maximal inhibitory concentration (IC_50_), were converted to the natural log value. In addition, we compiled 458 genes from a previous study that react with sensitivity or resistance to 130 anticancer drugs [44].

### Inferring putative cancer genes

We wrote a computer program (R script) to analyze all the pocket mutations and to obtain the number of missense mutations inside each pocket region of each protein. The script also calculates the number of missense mutations outside of the pocket region(s) of each protein by subtracting the pocket mutations from the somatic mutation dataset. This R script is provided in Additional file 2. In this study, the null hypothesis is that there is no significant association between the two category variables (pocket mutations versus non-pocket mutations). The alternative hypothesis of our computational approach is that if a gene has more somatic mutations in its protein pocket region in comparison to its non-pocket region (background mutations), this gene will more likely be cancer-related. We defined a background mutation as the total number of missense mutations in the non-pocket regions of all proteins (369 unique proteins, Additional file 1: Table S1). Then, we performed Fisher’s exact test, based on numbers in a 2 × 2 contingency table (Additional file 3: Table S2) for each protein. To identify the proteins that were significantly enriched with missense mutations in pocket regions versus at random, we required that the proteins have an adjusted *P* value (false discovery rate, FDR) of less than 0.1 after applying the Benjamini-Hochberg correction for multiple testing [45]. We performed the abovementioned Fisher’s exact test for each protein harboring pocket mutations in all cancer types (that is, pan-cancer) and again on each of the top 10 cancer types measured by the largest number of somatic mutations in the pocket regions. All statistical analyses (for example, Fisher’s exact test, Wilcoxon test, and Benjamini-Hochberg correction) were performed using the R platform (v3.0.1, [46]). All R codes used in this study are publicly available (Additional file 2).

### Kaplan-Meier survival analysis

To validate our results, we collected mRNA expression profiles and clinical annotation data of patients from the TCGA website [47]. Here, we used the mRNA expression profiles of three cancer types: lung adenocarcinoma, colon adenocarcinoma, and skin cutaneous melanoma. The RSEM (RNA-Seq by Expectation Maximization) values of mRNA [48] were used as the gene expression level measure. All *P* values were performed using a log-rank test. Notably, for the patients of lung and colon adenocarcinoma, 2,000 day (above 5-year) survival rates were used.

## Results

### Overview of somatic mutations in protein pocket regions

We mapped 1,195,223 cancer-related somatic mutations onto a set of 5,371 single-chain proteins with pocket region annotations in the PDB format. The SIFTS project provided mapping information for the genomic coordinates of somatic mutations and the sequence coordinates of PDB pockets. The final list was comprised of 2,262 unique somatic mutations in the pocket regions of 369 unique human proteins (see Additional file 1: Table S1 and Figure 1B).

We first examined the protein pocket region mutations at the sequence level. Among the 2,262 somatic mutations in the pocket regions, 1,603 (70.9%) were missense mutations, followed by 467 silent mutations (20.6%) (Figure 1B). Only a small portion of these mutations were nonsense mutations (115, 5.1%), which likely truncate protein sequences. The top 10 frequently mutated genes measured by missense mutations in the pocket regions were *PIK3CA*, *HRAS*, *CRP*, *AKT1*, *NCF1*, *NCAM2*, *VWF*, *ETV6*, *IFNB1*, and *KDM5C* (Figure 1C). It is worth noting that five of these genes (*PIK3CA*, *HRAS*, *AKT1*, *ETV6*, and *KDM5C*) are known to play important roles in cancer and are CGC genes (that is, experimentally validated cancer genes [35], see Methods). The average number of mutations in a pocket region(s) per protein is 6.1 (2,262/369) with 4.3 missense mutations on average per protein (1,603/369). For cancer types, somatic mutations in the pocket regions were more frequently observed in uterine, skin, colon, stomach, breast, lung adenocarcinoma, head and neck, lung squamous cell, and bladder cancer than in other types (Figure 1D).

### Hotspot amino acids measured by missense mutations in pocket regions

**Figure 2:**
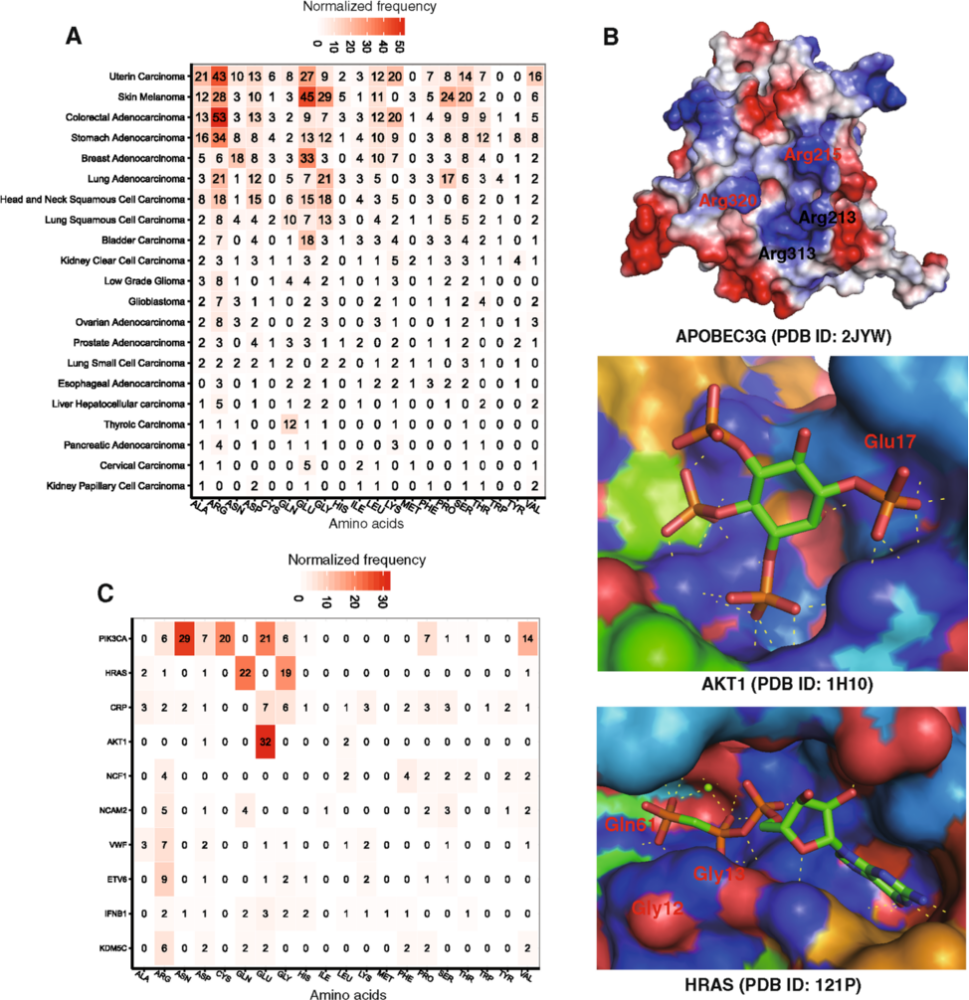
**The amino acid mutation spectrum in the pocket regions across 21 cancer types. (A)** The missense mutation spectrum of 20 amino acids in the pocket regions across 21 cancer types. **(B)** The detailed three-dimensional (3D) structures showing critical pocket mutations in three genes (*APOBEC3G*, *AKT1,* and *HRAS*). The PDB files were downloaded from the PDB database (http://www.rcsb.org/, accessed on 1 February 2014), and 3D pictures were prepared using software PyMOL (http://www.pymol.org/). **(C)** The missense mutation spectrum of 20 amino acids in the pocket regions of the top 10 frequently mutated genes.

We provided a catalog of amino acids involved in known somatic mutations within the pocket regions of each cancer type. This resource allows us to explore the features of somatic mutations, such as hotspot-mutated amino acids in the pocket regions and their underlying mutational processes. We examined the hotspot amino acids altered by somatic mutations across 21 cancer types using COSMIC and TCGA data. Figure 2A shows the spectrum of amino acid changes. We found that arginine (Arg) is a hotspot amino acid with a high frequency of somatic mutations in pocket regions across multiple cancer types, including uterine, skin melanoma, colon, stomach, head and neck, and lung cancers (Figure 2A). For example, Arg is attributed to the APOBEC family of cytidine deaminases [49]. APOBEC3G is a member of the polynucleotide cytosine deaminase gene family, which plays important roles in anti-viral immunity and cell cycles. As shown in Figure 2B, four arginine residues (Arg213, Arg215, Arg313, and Arg320) brim concave active sites in the APOBEC3G catalytic domain (PDB ID: 2JYW). Previous studies showed that these four Arg plays important roles in anti-viral immunity and cell cycles [50,51]. Besides Arg, glutamic acid (Glu) is another frequently mutated amino acid in the pocket regions of multiple cancer types, including uterine carcinoma, skin melanoma, breast adenocarcinoma, and bladder carcinoma. For example, *AKT1* is an important oncogene and plays a critical role in many cancer types [52,53]. Glu17 on protein AKT1 plays an important role during ligand-binding (PDB ID: 1H10) [53], which is a highly frequent, mutated residue in multiple cancer types, including breast, skin melanoma, lung, and colon cancers (Figure 2B and Additional file 1: Table S1). Furthermore, we examined the hotspot-mutated amino acids for the top 10 mutated genes (Figure 2C). Arg and Glu were frequently mutated amino acids on *PIK3CA*, *NCF1*, *AKT1*, *NCAM2*, *VWF*, *ETV6*, and *KDM5C*. Additionally, the asparagine (Asn), glycine (Gly), and glutamine (Gln) were frequently mutated in *PIK3CA* and *HRAS*. For example, Gly12, Gly13, and Gln61 were frequently mutated amino acids in the HRAS pocket (Figure 2B and Additional file 1: Table S1).

### Genes harboring pocket mutations were enriched in annotated cancer genes

**Figure 3:**
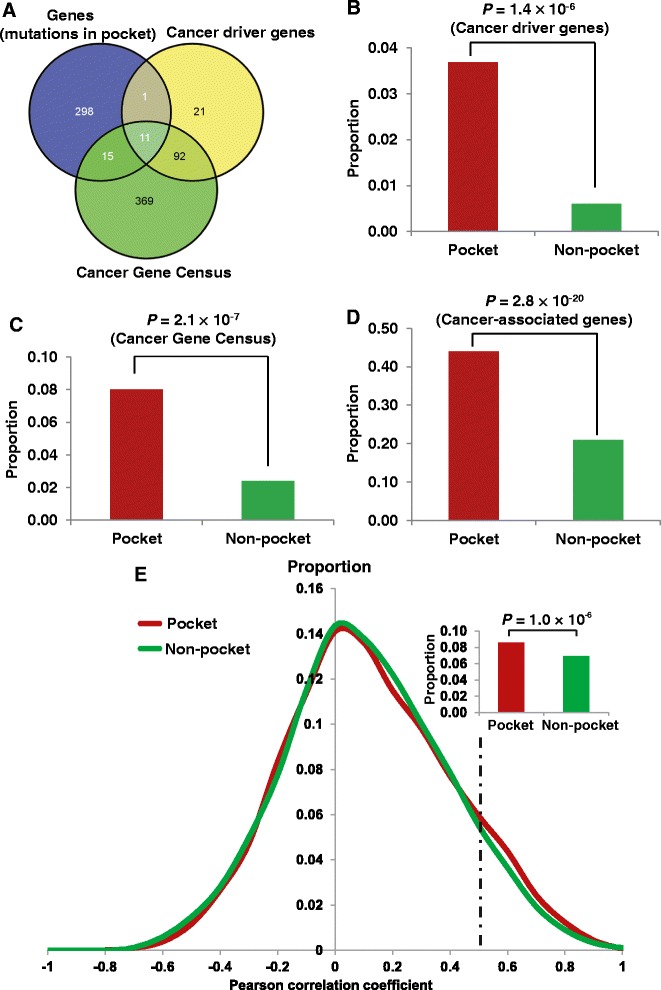
**The enrichment analyses of genes harboring pocket mutations in cancer genes and a co-expressed protein interaction network (CePIN). (A)** Venn diagram of genes harboring pocket mutations, cancer driver genes, and Cancer Gene Census (CGC) genes. **(B)** Genes harboring pocket mutations were enriched in cancer driver genes. **(C)** Genes harboring pocket mutations were enriched in CGC genes. **(D)** Genes harboring pocket mutations were enriched in cancer-associated genes. **(E)** Genes harboring pocket mutations tended to be highly co-expressed in CePIN. The *P* value was calculated using Fisher’s exact test. The detailed data regarding statistical analysis are provided in Additional file 4: Table S3 and Additional file 5: Table S4.

There were 1,603 missense mutations in the pocket regions of the proteins encoded by 325 genes. Among these 325 genes, 12 were cancer driver genes and 26 were CGC genes (Figure 3A, see Additional file 4: Table S3). We found that genes harboring pocket mutations were significantly enriched in cancer driver genes (*P* = 1.4 × 10^-6^, Fisher’s exact test, Figure 3B). Similarly, those genes harboring protein pocket mutations were more enriched in CGC genes (*P* = 2.1 × 10^-7^, Figure 3C) and cancer-associated genes (*P* = 2.8 × 10^-20^, Figure 3D and Additional file 4: Table S3) than in genes harboring non-pocket mutations (see annotated cancer gene details in Methods). Collectively, somatic mutations located in protein pocket regions tended to be associated with cancer genes. Caution should be taken that the analysis here might be influenced by incompleteness of protein structural data and somatic mutation profiles, as well as by the special cancer research interest of mutations in pocket regions.

### Genes harboring pocket mutations tended to be highly co-expressed in CePIN

To further explore the functional roles of pocket mutations on network level, we investigated the gene co-expression distribution for gene-gene pairs harboring pocket mutations. The PCC value of each gene co-expression pair was calculated from the microarray gene expression data of 126 normal tissues [40], as done in our previous study [41]. We mapped the PPC value onto a comprehensive protein interaction network (PIN) to build a CePIN (see Methods). This CePIN contained 90,705 PPI pairs connecting 9,945 proteins (Additional file 5: Table S4). Here, we defined a pocket PPI as one or two proteins in a PPI pair that harbors protein pocket missense mutation(s). In CePIN, we found 7,849 PPI pairs that connect proteins with pocket mutations. In this study, we designated those PPI pairs as functionally similar when the PCC value was more than 0.5, as in a previous study [54]. As shown in Figure 3E, pocket PPI pairs were more enriched in functionally similar PPI pairs (higher gene co-expression) in comparison to non-pocket PPI pairs (that is, neither of the two genes in a pair had pocket mutations) (*P* = 1.0 × 10^-6^, Fisher’s exact test). Detailed data regarding our statistical analysis were provided in Additional file 5: Table S4). Collectively, those genes harboring pocket mutations tended to be highly co-expressed in CePIN, implying their crucial functional roles through network perturbations [8,12].

### Inferring putative cancer genes

**Figure 4:**
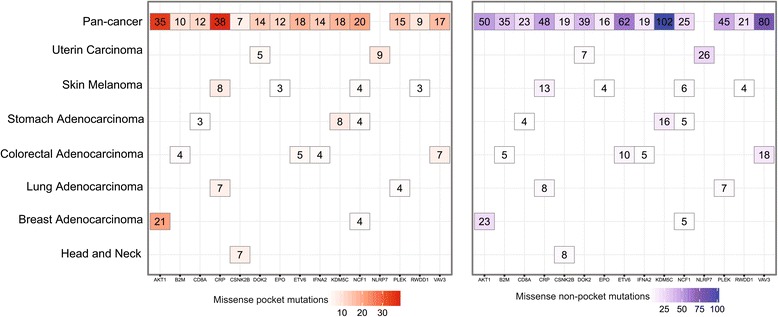
**The heat map of genes harboring somatic mutations that were significantly enriched in protein pocket regions.** Genes in each of the cancer types with an adjusted *P* value <0.1 are displayed and colored according to their frequency of missense mutations in the pocket regions (missense pocket mutations) versus in the non-pocket regions (missense non-pocket mutations). The detailed data are provided in Additional file 3: Table S2.

**Figure 5:**
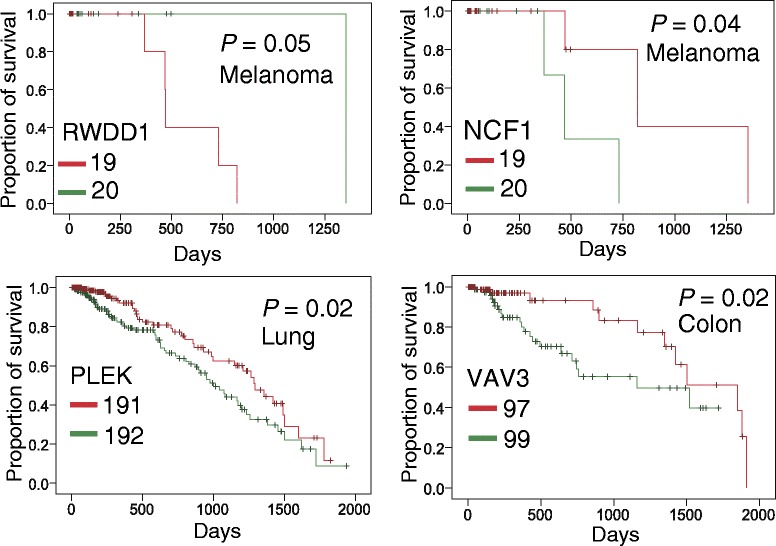
**Kaplan-Meier survival curves for four putative cancer genes identified by the statistical framework.** Patients were grouped into lowly (green) and highly (red) expressed groups based on the median expression levels of genes in skin cutaneous melanoma (melanoma), lung adenocarcinoma (lung), and colon adenocarcinoma (colon). The *P* values were performed using Mantel-Cox Log Rank test.

Our hypothesis stated that if a gene had more somatic mutations in its protein pocket region, this gene would more likely be cancer-related (Figure 3). In our pan-cancer analysis (21 cancer types), we found that 83 genes harboring somatic mutations were enriched in protein pocket regions (FDR <0.1, see Additional file 3: Table S2). Among the 83 genes, 44 were known cancer-associated genes [36]. For example, in our study *HRAS* (*P* = 5.0 × 10^-46^), *AKT1* (*P* = 9.5 × 10^-26^), *PIK3CA* (*P* = 5.5 × 10^-5^), *B2M* (*P* = 6.7 × 10^-4^), and *KDM5C* (*P* = 3.5 × 10^-3^) were predicted to be putative cancer genes using Fisher’s exact test and evidently designated as cancer driver genes according to the 20/20 rule [3]. To identify new cancer genes, we predicted several putative cancer genes in uterine, skin melanoma, colon, stomach, lung, head and neck, and breast cancers, respectively (Additional file 3: Table S2), since these cancer types have more somatic mutations in COSMIC database and TCGA. For skin melanoma, somatic mutations in four genes were significantly enriched in their protein pocket regions (Figure 4), including *CRP* (*P* = 2.2 × 10^-6^), *NCF1* (*P* = 6.3 × 10^-4^), *EPO* (*P* = 2.2 × 10^-3^), and *RWDD1* (*P* = 2.2 × 10^-3^). To further validate the predicted genes in melanoma, we performed a Kaplan-Meier overall survival analysis. We found that melanoma patients with high expression of *RWDD1* had a weak trend towards poor survival rates (*P* = 0.05, Figure 5). In another case, the low expression of *NCF1* was associated with poor survival rates in melanoma patients (*P* = 0.04). Collectively, *RWDD1* and *NCF1* [55] are two putative candidate targets for melanoma treatment. Further investigation on their roles is warranted.

For uterine carcinoma, the somatic mutations on two genes were significantly enriched in protein pocket regions: *DOK2* (*P* = 1.1 × 10^-4^) and *NLRP7* (*P* = 3.2 × 10^-4^). A previous study revealed that the loss of *DOK2* induces carboplatin resistance in ovarian cancer through the suppression of apoptosis [56]. Moreover, *DOK2* was found to act as a potential tumor suppressor in human breast cancer [57]. Ohno *et al.* [58] reported that the expression of the NLRP7 protein tend to be associated with poor prognosis in endometrial cancer tissues. Thus, our statistical framework could effectively predict known cancer genes in uterine carcinoma. For colon adenocarcinoma, the somatic mutations in four genes were significantly enriched in protein pocket regions: *B2M* (*P* = 3.1 × 10^-4^), *IFNA2* (*P* = 3.1 × 10^-4^), *VAV3* (*P* = 6.6 × 10^-4^), and *ETV6* (*P* = 1.0 × 10^-3^). Among them, *VAV3* is the member of the *VAV* family of Rho GTPas nucleotide exchange factors, and it reportedly has been involved in tumor progression and metastasis [59,60]. Notably, we found that somatic mutations of colorectal cancer were enriched in the VAV3 pocket region. Interestingly, colon cancer patients with downregulated *VAV3* expression were observed to possess significantly poorer survival rates (*P* = 0.02, Figure 5). We found that two genes in lung adenocarcinoma had enriched mutations in their pocket regions: *CRP* (*P* = 4.9 × 10^-7^) and *PLEK* (*P* = 2.1 × 10^-3^). Allin and Nordestgaard [61] reported that elevated circulating levels of CRP were associated with an increased risk of lung cancer. Again, we found that a low expression of the *PLEK* gene was associated with poor survival rates in lung cancer patients (*P* = 0.02, Figure 5). *PLEK* gene expression was reported to play a potential role in blocking neoplastic transformation [62]. Taken together, our protein structure-based approach appears effective in the identification of new putative cancer genes for future cancer biology studies.

### Case study: identification of new putative biomarker for anticancer drug sensitivity

**Figure 6:**
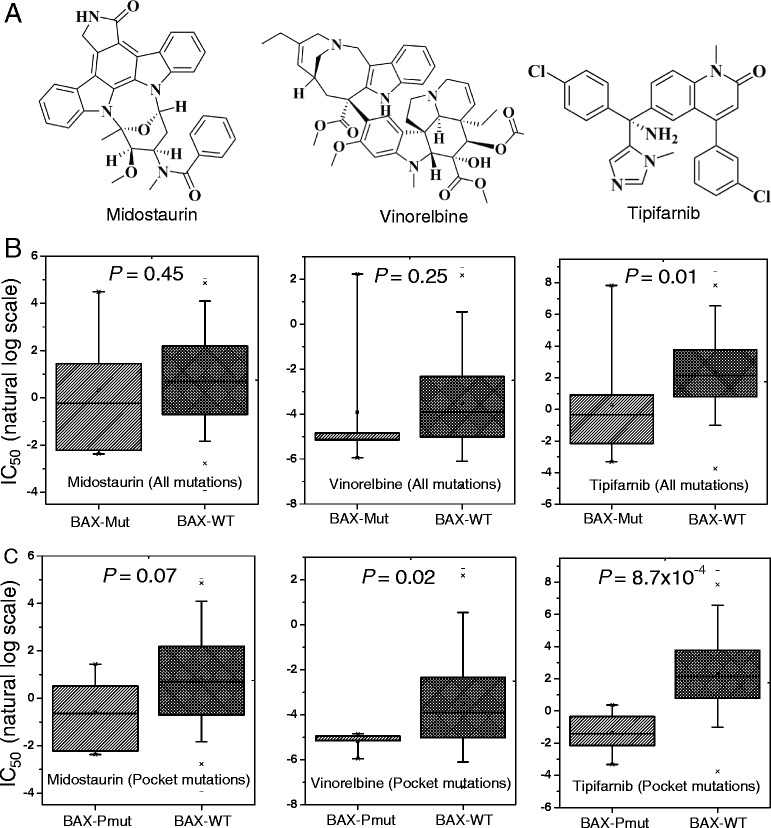
**Anticancer drug sensitivity analysis. (A)** The two-dimensional chemical structures of three anticancer drugs: midostaurin, vinorelbine, and tipifarnib. **(B)** The distribution of the half maximal inhibitory concentration (IC_50_, natural log scale) values for *BAX* gene-mutated cancer cell lines (BAX-mut) versus *BAX* gene wild-type cancer cell lines (BAX-WT) using all of the *BAX* gene’s mutation profiles. **(C)** The distribution of (IC_50_, natural log scale) values for BAX pocket mutated cancer cell lines (BAX-Pmut) versus BAX wild-type cancer cell lines (BAX-WT) using BAX protein pocket mutations only. The *P* values were calculated by the Wilcoxon test.

Identifying anticancer drug response markers through computational methods is highly promising for cancer precision therapy [63]. In this study, we sought to evaluate the putative drug sensitivity genes by incorporating drug pharmacological data, protein pocket information, and cancer cell line mutation profiles from the CCLE. We mapped 64,000 missense mutations and frameshift-inducing indels in 1,659 genes onto the protein pocket regions across approximately 1,000 different cancer cell lines. A total of 104 missense mutations and 36 frameshift indels were mapped in the pocket regions of 34 proteins. Next, we compiled 458 genes that displayed drug sensitivity or resistance to 130 anticancer drugs [44]. Our statistical analysis indicated that the genes harboring pocket mutations were enriched within anticancer drug response genes (*P* = 4.3 × 10^-7^, Fisher’s exact test, see Additional file 4: Table S3). Here, we provided an example (*BAX* gene) of identifying putative biomarker for anticancer drug responses. The *BAX* gene had the highest number of cancer cell line mutations in the pocket regions (PDB ID: 1F16). We first examined the *BAX* gene on vinorelbine, an anti-mitotic chemotherapy drug that is approved for breast cancer and non-small cell lung cancer treatment by the U.S. Food and Drug Administration (FDA). We divided the cancer cell lines into two subgroups: *BAX* gene mutated (*BAX*-mut) and *BAX* gene wild-type (*BAX*-WT), using all of *BAX* gene’s somatic mutation profiles. We found that the IC_50_ (natural log scale) of *BAX*-mut versus *BAX*-WT cancer cell lines on vinorelbine was not significantly different (*P* = 0.25, Figure 6B). Then, we divided the cancer cell lines into two subgroups: *BAX* pocket mutated (*BAX*-Pmut) and *BAX* wild-type (*BAX*-WT) using the BAX protein pocket somatic mutation profiles. Interestingly, the IC_50_ value of the *BAX*-Pmut cancer cell lines harboring protein pocket mutations on vinorelbine was significantly lower than that of *BAX*-WT cancer cell lines (*P* = 0.02, Wilcoxon test, Figure 6C). Similar patterns were observed when we examined the other two drugs: midostauin and tipifamib (Figure 6C). This example, plus the general patterns we identified, suggested that our integrative approach using protein pockets, somatic mutation, and drug pharmacological information is promising to identify anticancer drug response biomarkers in the emerging era of cancer precision therapy.

## Discussion

Recently, several large-scale cancer genome sequencing projects, such as the TCGA and ICGC, have released genomic landscapes of human cancer genomes, especially somatic mutations [1,2]. Such landscapes consist of a small number of ‘mountains’ (genes altered in a high percentage of tumors) and a much larger number of ‘hills’ (genes altered infrequently) [3]. Identifying the functional roles of the large volume of somatic mutations in hills is important for our understanding of how they function in tumorigenesis and tumor progression. Several recent studies have attempted the structure-based prioritization of functional mutations in cancer [11,21,22]. However, few have specifically explored the spectrum of somatic mutations in protein pocket regions. In this study, we developed a protein structure-based computational approach to explore the biochemical and structural roles of somatic mutations during tumorigenesis through the integration of large-scale somatic mutation profiles onto protein pocket regions.

The rationale of our computational approach is that if a gene has more somatic mutations in its protein pocket region, it is likely to be cancer-related. To test this hypothesis, we used three complementary methods: (1) cancer gene enrichment analysis: we found that genes harboring somatic mutations in their protein pocket regions were significantly enriched with cancer genes; (2) functionally similar pair enrichment analysis in co-expressed protein interaction networks: genes harboring somatic mutations in their pocket regions tended to be highly co-expressed in co-expressed protein interaction networks; and (3) anticancer drug response gene enrichment analysis: genes harboring somatic mutations in their protein pocket regions were more likely to be drug-sensitive or drug-resistant. Put together, somatic mutations located in protein pocket regions may be enriched with ‘actionable mutations’, and through their interactions drive tumorigenesis and alter anticancer drug treatment. To demonstrate the potential value of our approach, we identified four putative cancer genes (*RWDD1*, *NCF1*, *PLEK*, and *VAV3*), whose expression was associated with poor survival rates in melanoma, lung, or colon cancer patients. Furthermore, in a case study using a protein pocket-based approach rather than a traditional mutation versus wild-type approach, we concluded that the *BAX* gene was related to three anticancer drug sensitivities. There are two types of molecular mechanisms to explain mutations in pocket residues are drug-resistant or drug-sensitive. (1) A drug binds to a protein that directly involves the mutation(s) in the pocket. For example, several independent studies found that the actionable mutations in the *EGFR* gene could activate *EGFR* by altering the ATP binding site, which ultimately leads to an enhancement of drug response to gefitinib [24,25]. (2) The pocket mutations affect protein function, which subsequently perturbs the network nodes in the drug target’s signaling pathways, leading to drug sensitivity or resistance. The second mechanism is in a ligand-independent manner [26]. Here, we did not find any direct evidence in that bcl-2-like protein 4 (encoded by *BAX*) is a target protein involved in ligand-protein binding with midostaurin, vinorelbine, or tipifarnib [64-66]. Thus, *BAX* gene may perturb the network nodes in the signaling pathways, ultimately contributing to midostaurin, vinorelbine, and tipifarnib sensitivity [41,67].

Of note, the somatic mutational landscape within a cancer genome bears the signatures of active mutational processes [49,68]. In this study, we provided a catalog of amino acids involved in known somatic mutations within pocket regions and across cancer types. Our systematic analyses revealed that two amino acids, Arg and Glu, were most frequently mutated (hotspot mutations) within pocket regions across multiple cancer types. Specifically, Arg mutations were attributed to the anti-viral immunity and cell cycles of *APOBEC3G* [50,51], which is consistent with previous mutational signature analysis study [49]. Several recent studies, such as *SpacePAC* [13], *iPAC* [15], and *GraphPAC* [16], identified mutational clusters in cancer by integrating somatic mutation data and protein structure information. In comparison with these studies, our protein pocket-based approach provides an alternative to identifying actionable mutations in the pocket regions that are attributed to tumorigenesis, and further, to anticancer drug responses. In summary, our protein pocket-based integrative analysis provides important insights into the functional consequences of somatic mutations in cancer.

There are several limitations in the current work. First, the somatic mutation profiles from both the COSMIC and TCGA are mixed with driver and passenger mutations. Second, our approach requires protein 3D structural information to accurately detect protein pocket regions. The current protein pocket information is far from complete and may be inaccurate, due to the feasibility of protein structures [69]. Although about 100,000 protein and nucleic acid structures have been curated in the PDB database, the human protein 3D structure information is still far from being sufficient. In the future, we propose to improve our work in the two following ways: (1) use the experimentally validated driver mutations and passenger mutations from Vanderbilt’s MyCancerGenome database [70] to investigate the functional roles of driver mutations *versus* passenger mutations in protein pocket regions and non-protein pocket regions, and (2) integrate homology modeling protein pocket information from other organisms, as well as protein interface information in protein interaction network [54], large-scale atomic-resolution protein network [71], and protein post-translational sites (for example, phosphorylation sites) [72], to deeply explore the functional consequences of somatic mutations altered protein function in cancer. Despite its limit in the scope of the current investigation, the data allowed us to systematically explore the roles of somatic mutations in protein function and drug binding/response through a protein pocket prioritization approach. As a proof-of-principle study, we demonstrated that the protein structure-based strategy is a promising approach to gain insight into the functional consequences of somatic mutations in cancer.

## Conclusion

Detecting actionable mutations that drive tumorigenesis and alter anticancer drug responses is in high-demand in molecular cancer research and cancer precision therapy. In this study, we developed a protein pocket-based approach by incorporating large-scale somatic mutation profiles into the protein pocket regions. We found that genes harboring somatic mutations in their protein pocket regions tended to be cancer genes and anticancer drug response genes, and they had a trend to be highly co-expressed in co-expressed protein interaction networks. Overall, somatic mutations located in protein pocket regions could be functional mutations in cancer, and play important roles during tumorigenesis and for anticancer drug responses.

In addition, we demonstrated the potential value of the protein pocket-based approach to uncover putative cancer genes. Several genes that we identified through our approach have multiple lines of evidence from experimental data in literature. Building from our approach, we identified four new putative cancer genes (*RWDD1*, *NCF1*, *PLEK*, and *VAV3*), whose expression profiles were found to be associated with poor survival rates in melanoma, lung, or colon cancer patients. Finally, we predicted several putative biomarkers for anticancer drug responses through the integration of cancer cell line mutation profiles and drug pharmacological data from the Cancer Cell Line Encyclopedia with protein pocket regions. In a case study, we illustrated that the *BAX* gene was associated with three anticancer drug sensitivities: midostaurin, vinorelbine, and tipifarnib. In summary, this pilot study provides a unique investigation of the functional effects and molecular mechanisms of somatic mutations attributed to tumorigenesis and anticancer drug responses. We anticipate that future work will help identify how critical somatic mutations in pocket regions alter protein function in cancer, including protein-protein interactions and drug binding.

## Additional files

Additional file 1: Table S1. List of 2,262 somatic mutations in pocket regions across 369 unique human proteins. Additional file 2:
**The R code used in the protein pocket-based integrative analysis.**
Additional file 3: Table S2. List of genes whose somatic mutations are enriched in protein pocket regions. Additional file 4: Table S3. The 2 × 2 contingency table for enrichment analyses of cancer genes and anticancer drug response genes (Figure 3B, C, and D). Additional file 5: Table S4. A co-expression protein interaction network that is used for statistical analysis in Figure 3E.

## Abbreviations

3D: Three-dimensional
CCLE: Cancer cell line encyclopedia
CePIN: Co-expressed protein interaction network
CGC: Cancer gene census
ICGC: International cancer genome consortium
InCa: Index of carcinogenicity
PCC: Pearson correlation coefficient
PDB: Protein Data Bank
PIN: Protein interaction network
PPI: Protein-protein interaction
TCGA: The cancer genome atlas
